# GPT is all you need

**DOI:** 10.3389/fpsyg.2025.1549755

**Published:** 2025-10-10

**Authors:** Yuwen Zhang

**Affiliations:** School of Aeronautical, Mechanical, Mechatronics and Electrical, University of Sydney, Sydney, NSW, Australia

**Keywords:** Generative Pre-trained Transformers (GPT), cognitive augmentation, Hybrid AGI, multi-modal AI, cognitive scaffolding, neuroplasticity and AI, AI-human interaction

## Abstract

The advent of Generative Pre-trained Transformer (GPT) models, exemplified by systems like ChatGPT, has begun to reshape how humans think, learn, and interact. This paper explores GPT's role as a cognitive scaffold, supporting structured thinking, conversational agility, emotional regulation, and interdisciplinary learning. Grounded in established psychological frameworks—Cognitive Load Theory, Social Cognitive Theory, and Zone of Proximal Development—this work proposes theoretical mechanisms through which GPT may influence cognition, including neuroplasticity, meta-cognition, and implicit learning. While these claims remain speculative, the paper outlines future research pathways for empirically testing GPT's long-term cognitive impacts. It also introduces the concepts of multi-modal GPT and Hybrid AGI, defined as human-AI symbiosis systems that may extend cognition through sensory integration and co-adaptive learning. Limitations such as hallucination, surface-level learning, and cognitive overreliance are critically examined, alongside practical recommendations for educators, users, and developers. By offering a conceptual foundation and forward-looking agenda, this paper aims to catalyze interdisciplinary dialogue on GPT's evolving role in human cognition and learning.

## 1 Introduction

The adoption of Generative Pre-trained Transformer (GPT) models has introduced profound changes in human-AI collaboration, with applications spanning education, healthcare, and daily problem-solving (Ng et al., 2024; Sallam, 2023; Lee and Chung, 2024). While GPT itself is a model that generates text based on input sequences, interactive systems such as ChatGPT enable real-time engagement, allowing users to leverage AI-driven language models for diverse cognitive tasks. Recent studies in cognitive psychology demonstrate that GPT-4 can simulate human-like emotional reasoning (Tak and Gratch, 2024) and even exhibit decision-making heuristics observed in human cognition (Suri et al., 2024). These findings suggest that human interactions with GPT models are not merely transactional but influence cognitive processes such as structured thinking, memory scaffolding, and problem-solving strategies (Grinschgl and Neubauer, 2022).

The development of GPT models traces back to OpenAI's introduction of the first Generative Pre-trained Transformer (GPT-1), which applied unsupervised learning to large-scale textual data. Successive iterations, including GPT-2, GPT-3, GPT-3.5, and GPT-4, progressively improved in fluency, contextual awareness, and reasoning capabilities. These advancements stem from the Transformer architecture, first introduced in Google's seminal paper, *Attention Is All You Need* (Vaswani et al., 2017), which revolutionized deep learning approaches to natural language processing. Each successive GPT generation expanded the model's ability to process longer contexts, generate more coherent responses, and incorporate multi-modal capabilities, such as GPT-4's integration of vision-based inputs. Other conversational AI systems, such as Google Gemini (DeepMind, 2024), Deepseek (Liu et al., 2025), and Grok-3 (Musk, 2025), share similar functionalities but are based on distinct architectures.

In this paper, GPT applications refer specifically to software built upon OpenAI's GPT models, including but not limited to ChatGPT (OpenAI, 2022). For clarity, the term “GPT interactions” will be used throughout to denote human engagement with GPT-based applications specifically. While the discussion focuses on GPT-based interactions, it is possible that similar cognitive effects extend to other large language model (LLM)-powered conversational AI systems, such as Google Gemini and Anthropic's Claude. However, given that different architectures may influence user interactions in distinct ways, this paper limits its scope to GPT-based applications, with the potential for future research to explore broader generalizations.

From a psychological perspective, human interaction with AI can be understood through established frameworks such as Cognitive Load Theory and Social Cognitive Theory. Cognitive Load Theory (Sweller, 1988) provides a framework for understanding how cognitive burden impacts learning efficiency. GPT applications may align with this framework by helping manage cognitive load—structuring and simplifying complex tasks to make information more accessible and manageable. For instance, GPT models synthesize large amounts of fragmented data into organized and digestible components, allowing users to focus on higher-order processes like critical analysis and synthesis. Social Cognitive Theory highlights the role of observational learning and self-regulation in shaping behavior (Bandura, 1986). By modeling structured and adaptive communication, GPT interactions encourage users to unconsciously adopt similar strategies, fostering enhanced conversational agility and problem-solving abilities.

In the short term, GPT applications assist users by structuring fragmented information, allowing them to focus on higher-order processes such as critical analysis and synthesis. These immediate and mid-term benefits include enhanced conversational agility and improved task-based problem-solving. With repeated engagement over time, users may begin to internalize GPT's structured reasoning patterns, leading to the development of meta-cognitive skills—such as self-monitoring, adaptive learning, and interdisciplinary thinking. This progression aligns with Vygotsky's Zone of Proximal Development (ZPD), where external scaffolds help learners move from supported performance to independent mastery (Vygotsky and Cole, 1978). These layered cognitive benefits raise important questions about how GPT interactions may evolve from momentary support to enduring cognitive transformation.

To explore these possibilities, the following section draws upon well-established psychological theories to provide a conceptual foundation for analyzing GPT applications' role in structured thinking, adaptive learning, and cognitive scaffolding. Examining these frameworks will serve as the basis for the Key Observations section, which outlines how these mechanisms manifest in real-world GPT interactions.

## 2 Mechanisms behind GPT models's cognitive effects

The transformative cognitive and behavioral changes observed in GPT interactions can be understood through well-established psychological mechanisms. These mechanisms explain how GPT applications serves as a facilitator of structured learning, adaptable thinking, and skill transfer, ultimately fostering both short-term improvements and long-term cognitive growth.

### 2.1 Cognitive scaffolding and the zone of proximal development

GPT applications, leveraging structured guidance, serve as a powerful foundation for AI-driven applications, with conversational systems like ChatGPT providing structured responses that support cognitive scaffolding. While GPT models generate language outputs based on probabilistic predictions, ChatGPT offers a dynamic interface through which users can iteratively refine queries, receive contextualized feedback, and engage in structured dialogue. This aligns with Vygotsky's seminal concept of the *Zone of Proximal Development* (ZPD), which delineates the range between what a learner can achieve unaided and what they can accomplish with external guidance (Vygotsky and Cole, 1978). Within this framework, GPT applications function as an adaptive external support that helps users bridge cognitive gaps, fostering both structured learning and skill acquisition. According to ZPD, learning occurs most effectively when individuals operate just beyond their current skill level but within a range where assistance is beneficial (Wood et al., 1976). GPT applications exemplify this process by offering structured responses, clarifying concepts, and modeling systematic problem-solving strategies. Over time, through repeated exposure and engagement, users internalize these cognitive structures, enabling them to transition from reliance on GPT applications's guidance to independent mastery. The mechanisms through which GPT applications facilitate cognitive scaffolding include modeling and structured thinking, guided problem-solving, and self-regulated learning (Ng et al., 2024). By presenting clear, structured explanations and step-by-step reasoning, GPT applications help users develop an internalized framework for approaching complex problems. These systems provide iterative feedback that refines user thinking, akin to the role of a human tutor offering graduated assistance. Additionally, GPT applications foster metacognitive awareness by prompting users to engage in reflective questioning and self-assessment, reinforcing essential skills for independent learning. This aligns with Bandura's (1977) self-efficacy theory, which posits that repeated successful experiences enhance confidence and autonomy in learning.

An example of this can be observed in academic research and learning. A student initially relies on a GPT application to generate structured outlines and synthesize relevant literature for a complex academic paper. Through continued interactions, the student internalizes these organizational strategies, gradually reducing reliance on the GPT application and demonstrating autonomous cognitive control—precisely what ZPD theory predicts (Vygotsky and Cole, 1978). This transition highlights how GPT applications serve not merely as information retrieval tools but as interactive cognitive partners that enhance structured reasoning and learning transfer.

To further investigate GPT applications' role within ZPD, future studies should adopt longitudinal approaches to track user interactions over extended periods, assessing how scaffolding influences cognitive independence. Experimental designs could include pre- and post-intervention studies measuring changes in problem-solving efficiency, adaptability, and the transferability of skills across different domains. Understanding these mechanisms in greater depth will provide empirical validation for GPT applications' potential as cognitive augmentation tools.

By operating within users' ZPD, GPT applications serve as transformative cognitive scaffolds, not merely providing direct answers but equipping individuals with structured reasoning skills that enhance independent cognitive abilities. Future interdisciplinary research should explore how these effects manifest across different learning contexts, from academic settings to real-world professional problem-solving. As GPT applications continue evolving, understanding their role in scaffolding cognitive processes will be crucial in optimizing their integration into educational and professional environments.

### 2.2 Cognitive load theory and adaptive scaffolding

Cognitive Load Theory (CLT) explains how the human brain processes and retains information during learning, emphasizing the importance of managing cognitive load for effective knowledge acquisition. Sweller (1988) identified three types of cognitive load: intrinsic load, related to the complexity of the material; extraneous load, caused by inefficient instructional methods; and germane load, which facilitates schema construction and learning. When applied to GPT applications, CLT suggests that AI-driven systems can optimize learning by reducing extraneous load and increasing germane load through structured guidance. GPT applications achieve this by filtering, summarizing, and organizing complex information, preventing users from being overwhelmed while enhancing cognitive processing.

Adaptive scaffolding plays a crucial role in managing cognitive load. Research on dynamic scaffolding has demonstrated that AI-assisted learning environments significantly improve knowledge retention by adjusting the level of assistance in real time, helping users transition from novice to expert-level cognition (Wu et al., 2017). This aligns with how GPT applications dynamically adapt to user input, providing explanations, restructuring information, and breaking down concepts to optimize learning efficiency. Such mechanisms parallel the principles of CLT, where external supports are tailored to cognitive needs, ensuring that learning remains within the user's cognitive capacity.

By leveraging these principles, GPT applications foster a progressive reduction in cognitive dependency, allowing users to internalize structured reasoning and problem-solving strategies. Future studies should explore how prolonged interactions with GPT applications affect cognitive load distribution and whether AI-assisted scaffolding leads to sustained improvements in users' independent learning capabilities.

### 2.3 Social cognitive theory and observational learning

Social Cognitive Theory (SCT) explains learning as a process that occurs through observation, imitation, and modeling (Bandura, 1986). A key mechanism of SCT is observational learning, where individuals refine their cognitive and communicative abilities by engaging with well-structured examples (Bandura, 1986). GPT applications align closely with this framework by providing structured demonstrations of reasoning, language use, and problem-solving strategies, allowing users to internalize cognitive patterns that enhance decision-making and adaptability. In addition, GPT applications serve as highly responsive models of structured reasoning, delivering clear, context-aware explanations that users can internalize and later apply independently.

One study by Kartika (2024) demonstrated how university students using AI-powered writing assistants, such as Google Gemini, improved their writing efficiency through targeted AI feedback. The AI chatbot provided students with structured corrections, grammar enhancements, and vocabulary suggestions, allowing them to iteratively refine their writing. This tailored feedback loop aligns with observational learning, as students not only corrected their errors but also internalized the AI's structured articulation, improving their ability to compose clear and cohesive text over time. The interactive nature of AI-driven writing support also enhanced student engagement and motivation, reinforcing the self-regulatory aspects of SCT.

Additionally, SCT emphasizes self-efficacy, wherein individuals develop confidence in their abilities through repeated successful experiences (Pintrich, 2000). GPT applications provide a safe, low-stakes environment for iterative problem-solving, enabling users to refine their approaches with immediate AI feedback. This process fosters adaptability, allowing individuals to transfer acquired skills across various domains, ultimately supporting interdisciplinary expertise. Empirical evidence supports this alignment; for example, a study found that AI-driven conversational models improved conversational skill mastery by 17.6% in simulated interactions, demonstrating how users can learn and apply structured communication strategies through observation and practice (Lin et al., 2024). These enhancements reinforce the role of AI as a cognitive scaffold, supporting users' self-regulation, adaptability, and confidence in decision-making.

While this empirical evidence supports the theoretical alignment between SCT and GPT applications, additional research is needed to fully quantify these effects over time. Future studies could explore how sustained GPT usage influences users' cognitive and behavioral adaptations, particularly in structured thinking and communication efficiency. Examining these dimensions longitudinally would provide deeper insights into AI's role in fostering meaningful cognitive development.

By framing GPT applications within Social Cognitive Theory, we highlight how AI serves as a transformative learning tool. GPT applications not only provide users with information but also facilitate structured cognitive development by modeling clear, adaptive reasoning and communication strategies. Future interdisciplinary research should further explore how AI-driven cognitive scaffolds contribute to real-world skill acquisition across different professional and educational contexts.

### 2.4 Critical limitations in GPT-assisted cognition

Despite their transformative capabilities, GPT models remain bounded by several critical limitations that constrain their reliability as cognitive partners. These challenges must be foregrounded to contextualize claims about cognitive augmentation and learning enhancement.

One of the most prominent limitations is *hallucination*—GPT's tendency to generate plausible-sounding but false or unverifiable information (Ji et al., 2023). This stems from the model's reliance on statistical pattern matching rather than grounded truth verification, making it especially problematic in high-stakes contexts like healthcare, law, or scientific research. Users may unknowingly integrate these hallucinated facts into their reasoning processes, introducing epistemic risk and eroding trust in the model's output.

Another key concern is *surface-level learning*. While GPT models can generate text that mimics deep understanding, their knowledge representations are ultimately shallow, lacking causal reasoning, abstraction, or long-term memory (Bubeck et al., 2023). This can lead to overestimation of the model's conceptual depth and a false sense of mastery in users who rely heavily on its outputs without critical reflection. As such, GPT's support for learning is best seen as scaffolding rather than substitution.

A third limitation lies in the risk of *cognitive overreliance*. As users increasingly delegate cognitive tasks—such as summarization, coding, or ideation—to GPT models, they may experience a gradual erosion of independent reasoning skills. This phenomenon, similar to automation bias in human-computer interaction, is especially concerning in hybrid systems where AI continuously adapts to user behavior. Without explicit boundaries and reflection mechanisms, GPT use may unintentionally displace core human cognitive functions rather than augment them.

Finally, GPT models lack contextual awareness beyond the session's input window, making it difficult for them to track goals, intentions, or longitudinal learning processes. This limitation restricts their effectiveness in educational or strategic domains that require sustained coherence across time. Efforts to integrate memory and personalization remain in early stages and carry their own risks, including privacy, bias reinforcement, and loss of serendipitous discovery.

Taken together, these limitations highlight the need for critical engagement with GPT systems—not as flawless tools, but as evolving collaborators that require human judgment, oversight, and intentional use. The remainder of this paper explores the ways GPT can augment cognition, but always with these foundational caveats in mind.

## 3 Key observations

In summary, GPT interactions present a dual impact on human cognition—offering significant enhancements while posing short-term challenges. By fostering structured thinking, conversational agility, emotional intelligence, and learning transfer, GPT applications serve as a powerful cognitive scaffold that empowers users to achieve deeper understanding and cross-domain adaptability. However, limitations such as cognitive overload, over-reliance, and surface-level learning underscore the need for balanced, intentional usage.

To fully understand these cognitive transformations, it is essential to examine the underlying psychological mechanisms that drive GPT applications's cognitive effects. The following section explores these mechanisms through established cognitive theories, including *Cognitive Load Theory, Social Cognitive Theory, and the Zone of Proximal Development*. These frameworks offer insights into how GPT applications facilitate learning, adaptation, and problem-solving, ultimately shaping users' cognitive development over time.

### 3.1 Structured thinking and analytical abilities

GPT applications, powered by GPT models, promote enhanced structured thinking and analytical abilities by providing users with structured responses that help break down complex ideas into manageable components. However, it is to be noted that the clarity and coherence of these responses depend on user input, as different prompts can yield varying levels of systematic reasoning and detail. This structured approach enables users to analyze and organize information more effectively, fostering critical thinking and decision-making. The cognitive benefits of this interaction align with Vygotsky's *Zone of Proximal Development* (ZPD), which emphasizes how external scaffolding supports learners in internalizing structured reasoning frameworks (Vygotsky and Cole, 1978). By engaging with GPT applications' logically sequenced responses, users gradually develop an improved capacity for organizing and synthesizing information

Beyond structured reasoning, GPT applications play a crucial role in cognitive load management. By consolidating fragmented information into coherent narratives, GPT allows users to focus on higher-order cognitive processes such as synthesis and evaluation rather than expending cognitive resources on organizing disparate data points (Sweller, 1988). However, GPT applications are not infallible; they can generate hallucinated content—incorrect, outdated, or contextually irrelevant information—especially when dealing with ambiguous or poorly framed queries. While studies on cognitive offloading support the role of AI in enhancing problem-solving efficiency (Risko and Gilbert, 2016), users must remain critical of GPT-generated outputs and verify key information to mitigate the risks of misinformation.

The practical applications of structured thinking facilitated by GPT applications extend across multiple domains. In research and writing, these AI-powered tools assist users in generating well-organized outlines, synthesizing academic literature, and refining arguments (Ng et al., 2024). Similarly, problem-solving benefits from GPT applications' ability to provide step-by-step guidance for complex challenges, fostering an analytical approach to multifaceted issues (Tapia-Mandiola and Araya, 2024). Within educational settings, GPT applications serve as interactive tutors that model structured reasoning and logical argumentation, helping students develop a more systematic approach to problem-solving (Mitchell et al., 2019).

While these benefits present compelling insights into GPT applications' role as cognitive scaffolds, they should be regarded as speculative, grounded in established psychological theory rather than direct empirical evidence. Although frameworks such as ZPD and anecdotal experiences provide conceptual support, there is a notable lack of large-scale empirical studies quantifying these effects. Future research should therefore focus on controlled experiments—for instance, classroom interventions or workplace trials—to assess whether repeated GPT use reliably enhances structured thinking, analytical reasoning, and decision-making across real-world contexts. The absence of such statistical evidence underscores the novelty of this perspective and highlights an opportunity for interdisciplinary research into AI-assisted cognition.

### 3.2 Conversational agility and multitasking

GPT interactions appear to enhance *conversational agility*—the ability to adapt communication style, tone, and content based on context. These outcomes are theoretically aligned with *Social Cognitive Theory*, which emphasizes learning through *observational modeling* (Bandura, 1986), but should for now be regarded as hypotheses rather than empirically confirmed effects. By exposing users to dynamic, context-aware responses, GPT applications help develop flexible communication strategies that naturally transfer into real-world conversations. Over time, repeated interactions encourage users to refine their tone and style across different settings, from professional correspondence to creative brainstorming (Fui-Hoon Nah et al., 2023). Additionally, GPT applications enable clear and concise expression, even in multi-faceted, fast-paced discussions (Akdilek et al., 2024).

Rather than asserting that GPT directly enhances cognitive multitasking, this effect can be understood as a logical outcome of cognitive offloading. Humans can delegate tasks to external tools, reducing mental resource allocation and enabling simultaneous focus on multiple cognitive demands. Research on cognitive offloading (Risko and Gilbert, 2016) supports this, demonstrating that offloading information to external aids lightens cognitive load. Furthermore, AI-assisted offloading allows individuals to “deploy their insights strategically instead of relying on memorized facts” (Grinschgl and Neubauer, 2022), potentially fostering improved multitasking capacity. By handling sub-tasks such as memory recall and structuring responses, GPT may reduce cognitive strain, allowing users to engage more effectively in complex, multi-faceted conversations.

Empirical evidence further supports the role of AI-assisted communication in enhancing efficiency. Brynjolfsson et al. (2023) analyzed the impact of AI-driven conversational assistants among 5,000 customer support agents, finding a 15% increase in productivity, particularly benefiting less experienced workers. Similarly, Schmidhuber et al. (2021) investigated AI-facilitated human-chatbot interactions in enterprise environments, reporting that AI-assisted users experienced lower cognitive load and improved productivity compared to traditional non-AI-based solutions. These findings highlight GPT application's potential as a tool that not only refines communication but also optimizes cognitive efficiency in demanding workspaces.

Despite these advantages, short-term challenges exist. The rapid responsiveness of GPT applications can contribute to mental stress, particularly during prolonged interactions that require sustained engagement with AI-generated outputs (Nizamani et al., 2024). The work suggests that frequent interactions with AI technologies correlate positively with cognitive improvements but also elevate stress levels and emotional strain, highlighting the risk of cognitive overload and mental fatigue when users over-rely on AI-assisted decision-making. Additionally, while AI-driven exchanges enhance efficiency and reduce extraneous cognitive load, over-reliance on AI-generated responses may also reduce mental engagement and cognitive stimulation, potentially impacting critical thinking and problem-solving skills over time (Dergaa et al., 2024). This underscores the importance of balancing AI-assisted communication with moments of cognitive rest and active engagement to prevent mental exhaustion and maintain cognitive adaptability.

Over the long term, repeated GPT interactions allow users to internalize effective conversational strategies, enhancing their ability to shift between different modes of communication with ease. The capacity to seamlessly transition between diverse topics, maintain clarity in high-demand discussions, and integrate structured dialogue into everyday interactions represents a critical step toward more effective, adaptable communication. As AI becomes increasingly embedded in professional and social contexts, understanding how users engage with these systems will remain essential in refining their role in human cognitive development.

It is important to note, however, that many of the claims in this section remain speculative, grounded in cognitive theory and supported by small-scale or indirect studies rather than large-scale empirical validation. Future research should therefore design controlled experiments—for example, tracking how repeated GPT-assisted interactions influence conversational agility and multitasking in professional and educational contexts—to determine whether these theorized benefits can be empirically verified.

### 3.3 Emotional intelligence and social engagement

Generative Pre-trained Transformer (GPT) applications' ability to model empathetic communication and context-sensitive responses may play a role in enhancing users' emotional intelligence. While GPT itself does not possess emotions, it is capable of producing structured and adaptive conversational cues that exhibit emotional intelligence in its interactions (Wang et al., 2024). These outcomes are theoretically grounded in Bandura's *Social Cognitive Theory*, which emphasizes learning through observational modeling (Bandura, 1986), but should for now be regarded as hypotheses rather than empirically confirmed effects. By consistently presenting emotionally intelligent responses, GPT serves as an effective model from which users can learn, reinforcing their own ability to engage in empathetic dialogue. Through repeated exposure to GPT-generated responses, users may observe and internalize strategies for expressing empathy, managing emotions, and navigating social contexts.

Empirical studies provide preliminary support for this learning effect. For instance, Sharma et al. (2022) demonstrated that AI-assisted conversational systems, where AI provides real-time guidance in human interactions, led to a 19.6% increase in conversational empathy between peers overall. While this study involved an augmented system (AI + human) rather than independent GPT use, it nonetheless illustrates how exposure to emotionally adaptive AI responses can foster improvements in human empathy. Similarly, Lin et al. (2024) found that AI-driven conversational agents reduced negative emotions like fear by up to 25% through structured simulations and feedback, providing scaffolding for emotional regulation and conflict resolution while enhancing interpersonal skills. These findings highlight promising directions where structured AI interactions may serve as training mechanisms for individuals seeking to refine their emotional and social competencies in real-world environments. Over time, users may refine their ability to recognize and adapt to emotional cues, engage in emotionally intelligent dialogue across diverse social settings, and strengthen interpersonal skills such as active listening and conflict resolution.

A relevant example of this potential can be seen in educational settings, where GPT applications are employed to assist teachers in navigating difficult classroom interactions. A teacher using GPT to draft responses for sensitive student interactions may, over time, internalize strategies for managing conflicts empathetically. GPT applications' ability to detect sentiment and adapt tone enables the teacher to enhance their own emotional awareness and engagement with students. Similarly, professionals in customer service, healthcare, and leadership roles may benefit from AI-assisted emotional intelligence training, reinforcing their ability to handle complex interpersonal challenges with greater sensitivity and confidence.

Despite these promising applications, many of the claims in this section remain speculative, supported by small-scale or indirect empirical findings rather than large-scale, longitudinal studies. Future research should examine whether sustained engagement with GPT applications produces measurable and durable improvements in emotional intelligence across diverse contexts, such as education, healthcare, and customer service. Such investigations would help distinguish temporary training effects from genuine, long-term cognitive, and emotional adaptation.

### 3.4 Cognitive overload and learning transfer

Generative Pre-trained Transformer (GPT) applications' real-time synthesis of large volumes of information can induce a state of continuous partial attention (CPA)—a cognitive behavior where individuals constantly scan and optimize incoming information to avoid missing anything (Rose, 2010). Unlike traditional multitasking, which is often motivated by productivity goals, CPA is a network-driven process where individuals strive to remain constantly connected to streams of new information. Linda Stone, who coined the term, described CPA as the tendency to function as a “live node on the network,” perpetually seeking novel input (Rose, 2010).

While CPA originally emerged in the context of digital communication, its effects may be amplified by AI-generated responses. GPT applications, by producing structured, context-aware outputs, can facilitate rapid sequential information processing, keeping users engaged in ongoing loops of AI-assisted content consumption. This heightened engagement can increase the risk of cognitive strain, leading to *cognitive overload*—a state where information-processing demands exceed individual capacity, hindering learning and decision-making (Sweller, 1988).

Research in neuroplasticity suggests that such cognitive challenges, while demanding, may also strengthen adaptive learning mechanisms. For example, Kleim and Jones (2008) showed that experience-dependent neural plasticity, driven by repeated cognitive challenges, is crucial for skill acquisition and adaptability. Similarly, Fissler et al. (2013) found that engaging in novel, demanding tasks enhances structural brain changes and cognitive function. These studies indicate that GPT-assisted cognitive overload could, hypothetically, reinforce neuroplasticity. Over time, users may internalize GPT's structured reasoning patterns and interdisciplinary adaptability, enabling them to synthesize knowledge across domains (Chauncey and McKenna, 2023).

Precedent exists in earlier AI-driven tutoring systems. Tools such as AutoTutor and Cognitive Tutors have been shown to improve critical thinking, problem-solving, and cognitive adaptability by engaging learners in structured, interactive tasks (Graesser et al., 2005; Aleven and Koedinger, 2002). Given GPT's far greater natural language and reasoning capabilities, it is reasonable to hypothesize that these effects could be magnified, with GPT interactions providing an implicit training ground for higher-order reasoning and cross-domain adaptability. For instance, a data analyst who frequently uses GPT to generate code snippets while synthesizing research may gradually transfer this structured problem-solving approach to other contexts, such as financial modeling or healthcare analytics.

Despite this promise, cognitive overload presents a clear trade-off: short-term fatigue versus potential long-term cognitive flexibility. Prolonged engagement with GPT applications may lead to temporary strain, especially during complex, multitasking interactions (Nizamani et al., 2024). Thus, while GPT applications may support knowledge transfer and interdisciplinary adaptability, they also carry risks of fatigue, over-reliance, and reduced independent reasoning if safeguards are not in place.

It is important to note that while principles of neuroplasticity and knowledge transfer are well established in cognitive science, their extension to GPT-assisted interactions remains hypothetical. The claims in this section should therefore be regarded as speculative, grounded in theory and supported by small-scale or indirect empirical findings rather than large-scale, longitudinal validation. Future research should examine whether sustained GPT use leads to measurable improvements in cross-domain learning and problem-solving—for example, through controlled experiments comparing pre- and post-GPT learning outcomes, or longitudinal studies assessing durable changes in cognitive flexibility. Such investigations will be crucial to clarifying whether GPT-driven cognitive overload functions primarily as a risk factor, a learning catalyst, or both.

## 4 Future directions

The transformative impacts of GPT interactions on cognitive processes, behavior, and skill acquisition raise important questions about its broader implications. This section outlines key areas for future research and practical considerations to enhance GPT applications' role as a tool for cognitive growth.

### 4.1 Multi-modal GPT interaction

The future evolution of GPT models lies in their integration with *multi-modal inputs*—technologies that enable AI to process and respond to diverse data types, including text, voice, images, video, and sensory signals. Multi-modal AI refers to systems capable of synthesizing multiple forms of data simultaneously to generate contextually rich and adaptive responses (Stryker, 2024). This mirrors human cognition, where vision, hearing, touch, and other senses operate in parallel to interpret the environment.

At present, multi-modal AI primarily engages with visual and auditory data. Advances in computer vision, speech recognition, and real-time language translation have made these interactions increasingly seamless and context-aware (Stryker, 2024). GPT models can analyze medical images, process spoken queries, and produce cross-lingual responses—all of which already enhance human-computer interaction.

Looking forward, future capabilities may expand beyond text, image, and audio to include neural, haptic (touch), and olfactory (smell) inputs. While still speculative, these modalities are backed by emerging technologies. For instance, brain-computer interfaces could allow direct communication between users and GPT systems, bypassing conventional input channels (Neuralink, 2025). Similarly, haptic feedback systems—such as soft robotic gloves that deliver kinesthetic force cues—have demonstrated improved immersion and precision in virtual environments (Jadhav et al., 2017). Electrotactile interfaces are also under exploration for fine-motor feedback in AR/VR contexts, though results remain mixed (Kourtesis et al., 2022).

When paired with GPT models, such technologies could create immersive learning environments for domains requiring physical precision, like surgery, sculpting, or sports training. However, realizing this integration at scale will require the development of large, structured haptic datasets—analogous to the text and image corpora used to train current GPT models.

Olfactory interfaces offer another emerging frontier. Wearable scent-generating devices have been successfully integrated into VR systems to deliver contextual smells, such as in art installations or chemistry simulations (Liu et al., 2023; Lin et al., 2025). While these applications are still experimental, they demonstrate the technological feasibility of real-time scent output. To enable GPT models to reason with or generate olfactory content, however, standardized smell databases and mappings—linking molecular profiles to human-perceived descriptors—would need to be developed and embedded into training pipelines.

In short, the hardware required to sense and deliver haptic and olfactory feedback is becoming available. The primary barrier now lies in curating the corresponding sensory datasets and developing frameworks for GPT models to process and respond to them meaningfully. Once established, these sensory signals could flow bidirectionally: users could send smell/touch input to GPT systems via sensors, and GPT could generate sensory outputs back to users via actuators or wearables. This bidirectional architecture may become especially relevant in later stages of GPT evolution, including the speculative development of human–AI symbiosis, discussed in subsequent sections.

These future-oriented modalities open compelling possibilities for multi-modal GPT systems to transform education, design, and embodied learning. A user interacting with a GPT-powered virtual kitchen, for example, could one day smell the ingredients being discussed, feel the texture of virtual dough through a glove, or receive real-time corrections on knife grip via haptic feedback—all while receiving natural language guidance. While such applications remain conceptual, the necessary components are beginning to emerge.

Nonetheless, practical and ethical challenges persist. The fusion of visual, auditory, tactile, olfactory, and neural data streams increases the risk of cognitive overload, potentially diminishing learning efficiency. Privacy concerns are especially pronounced when dealing with brain-computer interfaces and bio-sensory devices, as these may capture deeply personal information (Yuste et al., 2017). Furthermore, aligning multi-modal GPT outputs with human cognitive rhythms remains an open design problem—effective learning hinges on intuitive, non-intrusive AI-human coordination.

In summary, multi-modal GPT interactions represent a promising next frontier in AI development. As sensory input/output devices mature and datasets expand, GPT systems may become capable of engaging users in deeply immersive, multisensory experiences. The integration of touch and smell alongside vision, audio, and text could significantly enhance the depth, adaptability, and impact of AI-mediated learning. However, translating technical feasibility into sustainable practice will require careful attention to cognitive limits, ethical safeguards, and the development of robust sensory corpora.

### 4.2 Expanding perspectives on hybrid AGI

The path to Artificial General Intelligence (AGI)—systems with human-level adaptability across cognitive domains—has often focused on standalone AI models achieving general-purpose intelligence. However, a promising alternative lies in human-AI integration, where intelligence emerges not from artificial systems alone but from the combined capacities of humans and machines.

This concept is typically referred to as Hybrid Intelligence, which describes systems where humans and AI collaborate to solve problems by leveraging complementary strengths. Hybrid Intelligence aims to enhance decision-making, creativity, and problem-solving by combining AI's computational speed and memory with human intuition, ethical reasoning, and contextual awareness. As Dellermann et al. (2021) describe, human-AI teams can outperform either component alone. Similarly, Krinkin and Shichkina (2022) propose co-evolutionary models grounded in cognitive interoperability between humans and machines.

Building on this foundation, this paper introduces the concept of Hybrid AGI as an evolutionary step beyond Hybrid Intelligence. While Hybrid Intelligence emphasizes cooperation for task-specific gains, Hybrid AGI refers to a distributed general intelligence system composed of both human and AI agents working in continuous partnership. Rather than striving for artificial systems to replicate all aspects of human cognition, Hybrid AGI integrates human minds as irreplaceable components of a broader, symbiotic cognitive system capable of general-purpose reasoning and adaptation.

This evolution is enabled by what we term Cognitive Symbiosis—a bidirectional process of co-adaptation where human and AI cognitive processes influence and reshape each other over time. In contrast to traditional AI tools, which operate independently of user cognition, cognitive symbiosis involves ongoing mutual learning. The AI system (e.g., GPT) adapts to the user's cognitive preferences, strategies, and feedback, while the human adjusts their reasoning and decision-making patterns in response to the AI's suggestions, structures, and memory scaffolds. Over time, this interaction forms a self-reinforcing loop that enhances both parties' capabilities.

This paper argues that cognitive symbiosis is not merely a byproduct of human-AI collaboration but the core mechanism that will define the next phase of general intelligence development. Hybrid AGI is thus not a stopgap on the path to autonomous AGI, but a final architecture in its own right—where general intelligence arises through sustained, adaptive collaboration between human and artificial cognition.

The preceding section explored how multi-modal GPT systems expand AI's capabilities through sensory modalities such as olfaction (smell) and haptics (touch). These technologies—while promising—depend on hardware such as scent emitters or robotic gloves, limiting their accessibility in everyday settings. Hybrid AGI, as conceptualized here, proposes an alternative paradigm: humans themselves become the sensory conduit. Rather than relying on embedded sensors, individuals perceive textures, scents, or emotional cues and communicate them through natural language or neural signals to AI. This transforms the human into an active sensor and interpreter—allowing GPT to adapt to subjective and context-rich human experiences without the need for external devices.

GPT models, as language-based AI, offer structured reasoning, externalized memory, and dynamic knowledge synthesis. Future developments—such as integration with neural interfaces or wearable haptic systems—could elevate GPT from a conversational tool to a full-spectrum sensory collaborator. While these integrations remain speculative, they are grounded in emerging experimental systems and research in brain-computer interfaces, wearable haptics, and cognitive augmentation. Their potential for Hybrid AGI warrants conceptual exploration despite limited empirical evidence to date. Viewed this way, Hybrid AGI becomes more than a passive assistant: it evolves into a real-time cognitive feedback system that accelerates learning, reconfigures reasoning strategies, and supports the continuous refinement of human intelligence.

The integration of GPT models into human workflows unlocks new possibilities across disciplines. In scientific discovery, hybrid systems can accelerate research by combining GPT's large-scale synthesis capabilities with human intuition, enabling novel hypothesis generation and cross-disciplinary insight. While progress in AI-driven knowledge extraction is well established, the direct impact of Hybrid AGI on discovery pipelines remains an emerging area of study. In creative collaboration, GPT models augment ideation in writing, visual design, and filmmaking (Mitchell et al., 2019), offering real-time scaffolding without displacing human agency. In complex decision-making domains such as healthcare and engineering, hybrid systems combine AI's precision and analytical scope with human ethical judgment and context sensitivity (Topol, 2019). Together, these applications demonstrate Hybrid AGI's potential as a broad-spectrum amplifier of human cognition across both analytical and creative domains.

While Hybrid AGI offers transformative potential, several critical challenges must be addressed to ensure its ethical, safe, and equitable development. One key concern is cognitive overreliance. Prolonged dependence on AI may erode independent human reasoning. As Hybrid AGI systems actively adapt to users, they risk replacing—rather than supporting—human problem-solving unless boundaries are clearly maintained. Another issue is trust and transparency. Continuous co-adaptation between human and AI makes decision processes harder to trace. Explainable AI methods must evolve to handle the dynamic, context-driven nature of Hybrid AGI outputs (Lipton, 2018). Ethical considerations also arise in domains where outcomes affect lives, such as medicine or law. Here, Hybrid AGI introduces tension between AI recommendations and human accountability. Additionally, unequal access to cognitive augmentation technologies may deepen existing inequalities in decision-making capabilities. As AI systems become increasingly embedded into thought processes, the line between personal cognition and machine-augmented reasoning may blur, raising complex questions about authorship, agency, and expertise. Finally, since Hybrid AGI learns from human behavior and simultaneously shapes it, feedback loops may reinforce cognitive biases or narrow intellectual diversity. Safeguards will be needed to ensure hybrid systems foster balanced and diverse thinking styles.

Hybrid AGI marks a fundamental shift from AI as a tool to AI as a cognitive partner. As these systems become more embedded in how humans think, learn, and create, their influence will grow both in scope and depth. Future research should explore the cognitive effects of prolonged AI augmentation, the dynamics of co-adaptation, and strategies for maintaining human oversight. Ultimately, the goal is not to replace human intelligence—but to cultivate a symbiotic partnership where human and artificial cognition evolve together toward higher levels of insight, creativity, and problem-solving.

### 4.3 Outcome-agnostic research framework

The development of GPT models necessitates a research paradigm that embraces outcome-agnostic methodologies. Unlike traditional AI research, which often pursues narrowly defined objectives, outcome-agnostic approaches encourage exploration of emergent properties and unintended applications, broadening the scope of discovery and innovation. This approach is particularly valuable in AI research, where the complexity and unpredictability of intelligent systems often lead to unexpected findings and applications (George, 2024). By not being constrained by specific goals, researchers can investigate novel questions and phenomena, laying the groundwork for future advancements that may not have been initially anticipated.

Outcome-agnostic research, also known as exploratory research, is characterized by its flexibility and openness to new ideas. In the context of GPT models, adopting such a framework allows for the investigation of a wide array of potential functionalities and interdisciplinary impacts. This includes not only enhancing language understanding and generation but also exploring applications in diverse fields such as cognitive science, healthcare, and the arts. A research model that prioritizes open-ended inquiry fosters an environment where serendipitous discoveries are more likely to occur, as researchers remain open to exploring the full spectrum of AI capabilities.

Implementing an outcome-agnostic research paradigm involves several core principles:

- **Exploratory modeling:** Allowing AI systems to generate novel insights and connections without predefined goals, fostering serendipitous discoveries (George, 2024).
- **Interdisciplinary collaboration:** Engaging researchers from diverse fields—such as psychology, neuroscience, and sociology—to identify unexpected synergies and impacts of GPT models.
- **Iterative refinement:** Continuously refining GPT models based on emergent feedback, ensuring that both positive and negative outcomes are systematically incorporated into future iterations.

By embracing these principles, outcome-agnostic research in GPT models can facilitate transformative applications across multiple disciplines. Rather than confining AI research to pre-established constraints, this approach unlocks new possibilities, extending AI's potential beyond traditional applications and fostering innovative solutions that contribute to a deeper understanding of artificial intelligence.

By embracing an open-ended research framework, GPT models can unlock transformative potential across domains. Historical breakthroughs, such as CRISPR and penicillin, highlight the value of unstructured exploration. GPT models, when developed under an outcome-agnostic framework, have the potential to reveal novel methodologies or frameworks in unexpected fields. By enabling autonomous pattern recognition and cross-disciplinary synthesis, AI can facilitate discoveries beyond its originally intended applications.

One area of unintended innovation is AI-driven rehabilitation, where virtual therapeutic systems powered by AI have opened new avenues for patient care, demonstrating the potential of AI to revolutionize traditional medical practices (Gonzlez et al., 2024). Similarly, in the arts, AI-generated creativity has led to the development of unique artistic expressions, challenging conventional notions of authorship and creativity (Van Hees et al., 2024).

Beyond these domains, outcome-agnostic AI models have also played an increasingly critical role in scientific discovery. Large-scale AI-assisted simulations are accelerating drug discovery, material science research, and environmental modeling, revealing structures and patterns that might have taken decades for human researchers to identify. In engineering, AI-driven optimization algorithms are enabling more efficient and sustainable designs, such as AI-enhanced aerodynamics for energy-efficient vehicles and bio-inspired structures in architecture.

Another promising direction is AI-human creative collaboration, where GPT models function as co-creators rather than mere tools. This extends beyond traditional AI-generated art, incorporating applications in film production, music composition, and interactive storytelling. AI models trained on vast creative datasets are now being used to generate adaptive narratives in video games, assist musicians in composing new melodies, and even produce entirely AI-directed short films.

By embracing an open-ended research approach, GPT models and other AI-driven frameworks can continuously expand their utility across domains, uncovering use cases that are not immediately apparent at their inception. Rather than being limited to predefined applications, AI can serve as a catalyst for novel discoveries, reshaping industries and fostering interdisciplinary innovation.

Outcome-agnostic research introduces risks that must be carefully managed. For example, open-ended exploration can lead to applications that raise ethical concerns, such as privacy violations or misuse in manipulative contexts (Yuste et al., 2017). In addition, outcome-agnostic research requires significant time and computational resources, which may not always align with immediate societal needs.

An outcome-agnostic research framework for GPT models aligns with the principles of exploratory science, prioritizing flexibility and interdisciplinarity to uncover transformative applications. While this approach entails challenges, such as resource demands and ethical risks, it remains essential for fostering innovation and ensuring that the full potential of GPT models is realized responsibly.

### 4.4 Risk mitigation in multi-modal GPT and hybrid AGI

The advancement of GPT models into multi-modal and Hybrid AGI systems introduces both opportunities and risks. While these technologies offer unprecedented capabilities in human-AI collaboration, they also present new challenges related to cognitive dependency, AI-human alignment, and ethical concerns in multi-modal AI applications. Unlike traditional AI risks—such as bias and misinformation—Hybrid AGI and multi-modal AI require proactive strategies to mitigate risks emerging from deep human-AI integration, evolving reasoning patterns, and expanding AI autonomy.

#### 4.4.1 Cognitive overreliance, fatigue, and AI-human misalignment

As Hybrid AGI systems become more sophisticated, prolonged reliance on AI for knowledge synthesis and decision-making may reduce users' capacity for independent reasoning and sustained attention. Multi-modal AI—integrating vision, speech, neural interfaces, and other sensory streams—further raises the risk of cognitive fatigue and automation complacency. To mitigate these effects, we recommend:

- **For educators:** Design learning activities that explicitly differentiate between AI-assisted and unaided reasoning. Encourage students to reflect on their cognitive processes when using AI-generated suggestions.
- **For users:** Periodically perform high-cognition tasks without GPT support to maintain intellectual resilience. Monitor signs of fatigue or overuse, such as passive acceptance of AI suggestions or declining curiosity.
- **For developers:** Implement adaptive scaffolding mechanisms that allow users to adjust levels of AI assistance, promoting engagement rather than dependence.

#### 4.4.2 Bias and misinformation in multi-modal AI

As GPT models incorporate capabilities across modalities—including image, voice, and even haptic inputs—they inherit biases from those datasets (Bender et al., 2021). In sensitive domains such as healthcare, education, or hiring, these biases can reinforce inequality or misinformation. To address this, practical safeguards include:

- **For developers:** Use cross-modal validation techniques, where reasoning from one input (e.g., text) is tested against others (e.g., image or speech).
- **For users:** Treat AI outputs as hypotheses rather than truths—verify against external knowledge sources or through human peer review.
- **For policymakers:** Enforce standards for dataset auditing and transparency across all sensory modalities, especially in regulated fields.

#### 4.4.3 Privacy and ethical concerns in sensory AI

The convergence of Hybrid AGI and multi-modal GPT—especially through neural interfaces and biometric-enhanced devices—amplifies risks to privacy, consent, and data sovereignty. These systems increasingly rely on intimate cognitive and physiological signals. To ensure ethical implementation:

- **For developers:** Employ secure-by-design architectures, with end-to-end encryption for brain-computer interface (BCI) or wearable haptic inputs.
- **For users:** Understand what types of cognitive or biometric data are being collected and retain the right to opt-out or anonymize inputs.
- **For institutions:** Establish review boards and ethics protocols specific to neural or sensory data collection, especially in education, healthcare, and defense.

Hybrid AGI and multi-modal GPT models represent a shift toward AI systems that do not simply process information but actively shape human workflows and cognition. As these technologies continue to evolve, risk mitigation must transition from static safeguards to dynamic oversight models that integrate user feedback, promote resilience, and protect human autonomy. Future research should explore how human-AI collaboration evolves over time, and how practical guidelines can help maintain the AI-human relationship as one of augmentation—not replacement.

## 5 Conclusion

The rapid adoption of GPT models has reshaped human cognition and behavior, acting as both a tool for immediate knowledge retrieval and a facilitator of long-term cognitive growth. Grounded in established psychological frameworks—including *Cognitive Load Theory, Social Cognitive Theory*, and *Vygotsky's Zone of Proximal Development*—this paper demonstrates GPT's potential role in supporting structured thinking, conversational agility, emotional intelligence, and interdisciplinary learning transfer. Evidence from related domains, such as AI-driven tutoring and cognitive scaffolding, provides preliminary support for these effects.

However, it is essential to distinguish between established insights and forward-looking hypotheses. Well-documented findings include the ability of AI systems to reduce cognitive load, model structured reasoning, and scaffold users' performance in immediate tasks. In contrast, claims about long-term neuroplastic effects, durable gains in emotional intelligence, or interdisciplinary cognitive transfer remain speculative. These possibilities are grounded in cognitive theory and small-scale empirical studies but require large-scale, longitudinal validation to be confirmed.

The potential of GPT models to act as *cognitive scaffolds* is promising but not without risks. Short-term limitations such as cognitive overload, over-reliance, and surface-level learning highlight the need for thoughtful design interventions, including adaptive pacing, reflective prompts, and phased scaffolding. Future research should also investigate whether sustained GPT interactions produce enduring cognitive benefits, or whether risks such as fatigue and reduced independent reasoning outweigh long-term gains.

Looking ahead, speculative directions include the integration of GPT with *multi-modal inputs*—such as virtual reality, neural interfaces, and sensory technologies—and its role in emerging *hybrid AGI systems*. While these remain conceptual at present, they highlight potential trajectories for future human-AI collaboration.

In conclusion, GPT models represent more than a technological innovation; they may serve as catalysts for cognitive transformation. Yet the extent of this transformation remains an open question. By explicitly distinguishing evidence-based claims from theoretical hypotheses, this paper underscores both the promise and the uncertainty of GPT's role in human cognition. Advancing this field will require rigorous empirical studies, interdisciplinary collaboration, and careful attention to ethical safeguards.

## Data Availability

The original contributions presented in the study are included in the article/supplementary material, further inquiries can be directed to the corresponding author.
